# Recent Advances in Combinations of TLC With MALDI and Other Desorption/Ionization Mass-Spectrometry Techniques

**DOI:** 10.3389/fchem.2021.771801

**Published:** 2021-12-16

**Authors:** Roman Borisov, Anastasiia Kanateva, Dmitry Zhilyaev

**Affiliations:** ^1^ A. V. Topchiev Institute of Petrochemical Synthesis, Russian Academy of Sciences, Moscow, Russia; ^2^ Peoples Friendship University of Russia (RUDN University), Moscow, Russia

**Keywords:** TLC, MALDI, ambient ionization mass spectrometry (AIMS), sorbents, derivatization

## Abstract

The combination of planar chromatography with desorption/ionization mass-spectrometry (MS) techniques provides chemists with unique tools for fast and simple separation of mixtures followed by the detection of analytes by the most powerful analytical method. Since its introduction in the early 1990s, thin-layer chromatography (TLC)/matrix-assisted mass spectrometry (MALDI) has been used for the analysis of a wide range of analytes, including natural and synthetic organic compounds. Nowadays, new desorption/ionization approaches have been developed and applied in conjunction with planar chromatography competing with MALDI. This review covers recent developments in the combination of TLC with various desorption/ionization MS methods which were made in recent several years.

## Introduction

The conjugation of thin-layer chromatography (TLC) and mass spectrometry (MS) is one of the promising hybrid analytical methods. Despite the seeming simplicity, TLC with different detection systems is actively used in analytical and organic chemistry for the express separation of various mixtures and preliminary quantification, for example, for the analysis of oil and petroleum products (Cebolla et al., 2016; Speight, 2020; Makowska and Pellinen, 2021), monitoring the progress of organic reactions (Cagniant et al., 2001; Siebenhaller et al., 2018; Sahaka et al., 2021), separation of dyes and inks (Barker et al., 2017; Mohammad et al., 2017; Sharma and Kumar, 2017; Miron-Merida et al., 2021), and monitoring target compounds in different media (Kumar et al., 2017; Qu et al., 2021).

The potential of TLC as a modern micro-scale separation method is based on a number of its convenient properties, such as large sample throughput and suitability for screening separations (Matheis et al., 2015). This method may also be used for preliminary testing of separation conditions for HPLC applications due to low-cost optimization of mobile phase composition and easy and fast change of the stationary phase. Sample preparation for TLC is also not such a rigorous step, as for other chromatographic methods due to the disposability of the TLC plates. One of the unique advantages of TLC is the possibility of long-term storage of the analytical information after the separation process, so the TLC plates with target compounds may be further conjugated to the other analytical methods, such as IR, FID, or MS detection. The main disadvantages of TLC are difficult quantification and rather limited possibilities of qualitative analysis with classical detection methods, especially while separating complex mixtures, and if the question of quantitative analysis remains open to this day, for example, Stanek et al., 2019 developed the TLC method for the express qualification and quantification of phenolic compounds and abscisic acid in honey, and succeeded with ∼10% relative error of the concentration of the target compound (depending on the substance) using densitometric detection; similar results were obtained by Hynstova et al. (2018) who performed the separation of carotenoids and chlorophylls in dietary supplements containing *Chlorella vulgaris* and *Spirulina platensis* using high-performance TLC (HPTLC) and many others; the qualitative determination of the target components in the composition of complex mixtures can be reliably performed using suitable detection systems, and one of the main analytical methods in this case is conjugation to MS. At the same time, MALDI, among all the MS methods, occurred to be one of the most suitable methods for direct conjugation to planar chromatographic methods, opening broad possibilities for the identification of components of complex “omics” mixtures, screening, especially for small molecules, and development of methodological approaches, including new stationary phases for TLC, which might be suitable for direct MS detection of the separated compounds. MERCK KGaA, for instance, produces the so-called MS grade TLC plates which possess some important advantages; these TLC plates are characterized by a very low MS background due to the control of MS-detectable impurities, and the stationary phase layer thickness has been decreased to 100 μm, which is twice lower than that of standard TLC plates, as it was found that layer thickness influences the efficiency of laser desorption from the plate surface (Hillenkamp and Peter-Katalinic, 2013; Griesinger et al., 2014). It also should be noted that though MALDI can also be combined with other separation techniques such as, for example, capillary electrophoresis and liquid chromatography, TLC appears to be more suitable for MALDI experiments because the plates can be directly introduced to the ion source without any additional interfaces.

The aim of the present article was to review the recent advances of the combined TLC-MALDI technique, including the dedicated stationary phases, matrixes, derivatization, and application of the method. The last part of the review is devoted to the conjugation of TLC and novel desorption/ionization mass-spectrometry techniques, such as DART, DESI, and some other non-commercial systems with significant potential.

## Thin-Layer Chromatography/Matrix-Assisted Mass Spectrometry

### Sorbents

In 2002, monolithic thin-layer sorbents based on silica gel containing no additional binder were introduced into laboratory practice by Merck (Germany). Together with the minimal thickness of the stationary phase layer (1–50 μm) providing improved separation properties, these TLC plates were found to be suitable to the direct coupling to different MS detection systems due to covalent binding of the stationary phase and, as a result, the low bleeding level. However, together with the advantages, a decrease in the thickness of the sorption layer also resulted in the decrease in the resolution due to the small elution pathway. The solution of the problem was in increasing of the separation efficiency, so the UTLC and HPTLC plates were taken into consideration. Increase of the efficiency allowed the increase of the sensitivity of the method, reaching about a dozen pmol per sample using UV detection and a few pmol with MALDI-MS.

Zhang et al. (2011) suggested the slides for ultrathin-layer chromatography (UTLC) which were prepared *via* coating nonporous silica particles, chemically modified with polyacrylamide, as 15 mkm films, on glass or silicon. The authors used the model mixture of three proteins, namely, myoglobin, cytochrome c, and lysozyme, for testing the separation ability of the prepared sorbent, and have received the nearly baseline resolution realized by the hydrophilic interaction chromatography mechanism. The sorbent demonstrated the HETP value of about 3 mkm, giving the opportunity to develop the separation efficiency more than 3500 TP per plate. Zhang et al. also varied the silica particle diameter, and tested the 900-, 700-, and 350-nm particles. The typical SEM image of a UTLC slide with a coating with silica nanoparticles demonstrates the smooth particles with narrow size distribution and optimal layer arrangement.

As it was found, the decreasing particle diameter improved the resolution but slowed down the separation. The plates with the optimized properties were used combined with MALDI-MS for the analysis of the proteins. MALDI for each protein was carried out at the region which was previously marked by the fluorescently labeled proteins under a microscope, and the mass spectrum for the center of each spot was recorded. The mass spectrum in each case agreed with the expected molar mass for each protein. The signal-to-noise ratio had an acceptable value, confirming the possibility of utilization of the approach.

In 2013, Svec et al. (Lv et al., 2013) suggested organic polymeric monolithic sorbents based on poly (4-methylstyrene-co-chloromethyl-styrene-co-divinylbenzene). The authors compared the different polymerization initiation approaches and have chosen the synthetic procedure based on the thermally initiated process. The photopolymerization was not suitable for aromatic monomers because the latter absorb light in the UV range, resulting in decreased efficiency of the process. F. Svec et al. demonstrated that monolithic thin layers with a small surface area were able to produce efficient separations of peptides and proteins together with their on-layer MALDI-TOF-MS. Separation and subsequent identification of the components of the mixture of (Met5) enkephalin, oxytocin, and melittin labeled with fluorescamine were performed by Svec et. al to demonstrate the plate efficiency. All three components were baseline-separated using 65% acetonitrile in 0.1% aqueous TFA solution as a mobile phase. The MALDI-TOF-MS spectra were obtained directly from the TLC plates based on the poly(4-methylstyrene-co-chloromethylstyrene-co-divinylbenzene) monolithic sorbent with layer thickness of 50 mkm from the analyte spots using cyano-4-hydroxycinnamic acid as a matrix. The authors found the MS spectra were characterized with a good signal-to-noise ratio, confirming the main point of the TLC-MALDI-MS conjugation. The article also contained the results for eight different plates and double spotting of peptide mixtures, which demonstrated good repeatability and reproducibility of stationary phase properties.

In 2015, Hu et al. presented a TLC-ESI-MS method for direct analysis of raw samples. The main idea for the conjugation of TLC and MS methods was in the fact that the TLC plate could serve as a medium for absorbing interfering substances, allowing the detection of target compounds with reduced matrix effects. The authors have adapted the conventional TLC plates with an aluminum base coated with silica gel stationary phase particles. The suggested mode of separation of the target compounds and interfering substances allowed the decrease of sample pretreatment. The described procedure was efficient in the direct analysis of samples with salts and detergents, and rapid detection and quantitation of the target analyte in raw biological fluids were possible.

**FIGURE 1:**
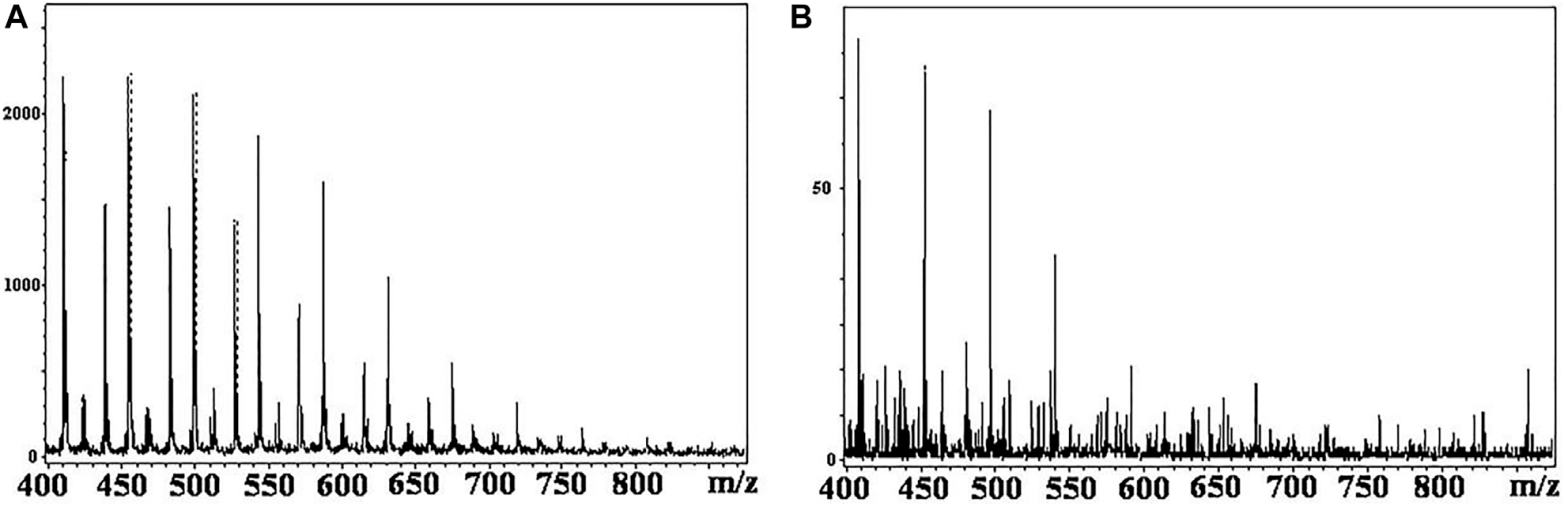
MALDI-MS spectrum for the PEG sample (1 mg/ml) received directly from the monolithic TLC plate without additional matrix compounds. **(A)** 29% (w/w) GMA and **(B)** 14% (w/w) GMA. Figure reprinted with permission from Kucherenko et al. (2018). Copyright (2018) WILEY-VCH Verlag GmbH & Co. KGaA.

However, the main difficulties for this combination are the application of the MALDI-MS matrix onto the TLC plate and subsequent ionization. Kucherenko et al. suggested TLC plates with an organic polymer monolithic layer to work out the problem (Kucherenko et al., 2018; 2019). The chemical nature of monolithic polymers provides an opportunity to incorporate into the polymeric layer the functional groups which could play a role of “matrix,” that is, to absorb and to redistribute the energy of laser pulse to ensure the soft ionization of the target compounds. At the same time, these functional groups will be covalently bonded to the polymer layer and will not result in a matrix cloud in the low molecular mass area of the mass spectra. Organic monolithic sorbents also have a highly developed surface area, allowing high-efficient separations due to the small size of the monolith domain structures. In the study by Kucherenko et al. (2018), the authors have developed the monolithic layers prepared by the copolymerization of ethylene dimethacrylate and glycidyl methacrylate, and deposited onto the surface of glass, and silicon plates were investigated as thin-layer chromatography separating media in hyphenated thin-layer chromatography–matrix-assisted laser desorption/ionization mass-spectrometry analysis. Varying compositions of the polymerization mixture and polymerization condition layers of different porosities and MALDI-MS compatibility were synthesized. The compatibility with MALDI-MS was tested using PEG, and it was demonstrated that layers prepared without glycidyl methacrylate are not compatible with MALDI-MS and do not allow obtaining any mass spectra. The best results were achieved with layers containing 19–29% glycidyl methacrylate. These layers allow mass spectra measurement without additional deposition of matrix compounds, supplying mass spectra almost “clean” in a low molar mass range (Figure 1).

### Matrixes

**FIGURE 2:**
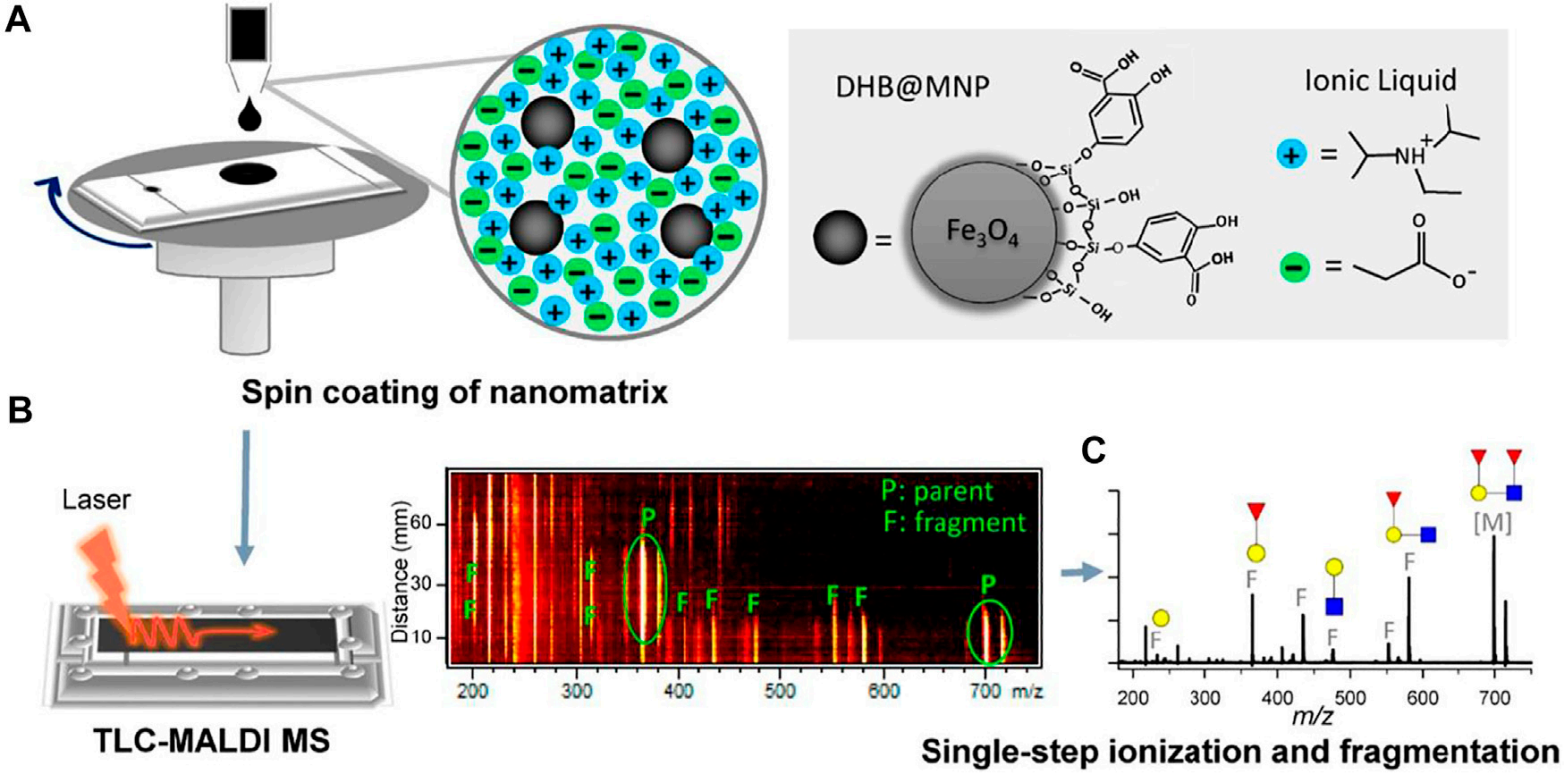
Analytical workflow of DHB@MNP-assisted TLC-MALDI-MS using a saccharide mixture as a model (Mernie et al., 2019). **(A)** Saccharide mixture was separated on a C18-modified TLC plate, and the DHB@MNP nanomatrix was deposited on the TLC surface by spin-coating with the ionic liquid. **(B)** Automated scanning of the entire TLC separation lane was performed to obtain TOF-MS spectra at different migration distances. **(C)** Finally, on-spot structural elucidation of the saccharides was performed based on the parent and fragment ions generated from DHB@MNP-assisted fragmentation in single TOF-MS. Figure reprinted with permission from Mernie et al. (2019). Copyright (2019) the American Chemical Society (ACS).

One of the important distinctions of MALDI-MS from other soft ionization methods is the key role of the matrix in the ionization process. There are a large number of matrix compounds suitable for the analysis of low molecular weight analytes (Calvano et al., 2018; Kobylis et al., 2021). However, recently, some new matrixes have been proposed for TLC/MALDI. Thus, Mernie et al. (2019) described a very promising approach allowing direct TLC/MS profiling of oligosaccharides. The method is based on using magnetic nanoparticles functionalized with the traditional matrix compound-2,5-dihydroxybenzoic acid (DHB). The nanoparticles were dispersed in an ion liquid and applied on the TLC plate using a spinning device (Figure 2). Desorption/ionization of oligosaccharides in such conditions proceeds simultaneously with their fragmentation, forming characteristic ions. The use of this composite matrix also causes increasing of peak intensities of analytes’ sodiated adducts and fragment ions.

Another example of the application of the inorganic matrix was proposed in the study by Fougère et al. (2018). The authors used core-shell silica-coated iron oxide magnetic nanoparticles as the matrix for the detection of flavonoid compounds by TLC/MALDI. The nanoparticle dispersion in ethanol was applied directly on the spots of interest, and MALDI mass spectra both in positive and negative modes were registered. The approach allowed the detection of anthocyanins in red wine, glycosylated derivatives of quercetin in apple juice, and polyphenols in rose flower extract.

It is important to note, however, that the use of solutions of matrixes can cause secondary chromatographic processes on TLC plates and, hence, analyte spot blurring. This problem can be overcome by using solvent-free matrixes such as graphite-assisted laser desorption/ionization (GALDI) (Han et al., 2011). The sample preparation process in this case looks very simple and convenient because the matrix can be applied on TLC plates by denoting spots with a simple pencil lead. A large-scale test of this approach made on a number of synthetic organic compounds confirmed its utility (Borisov et al., 2014). However, the authors underlined that TLC/GALDI requires rather energies of the laser.

**FIGURE 3:**
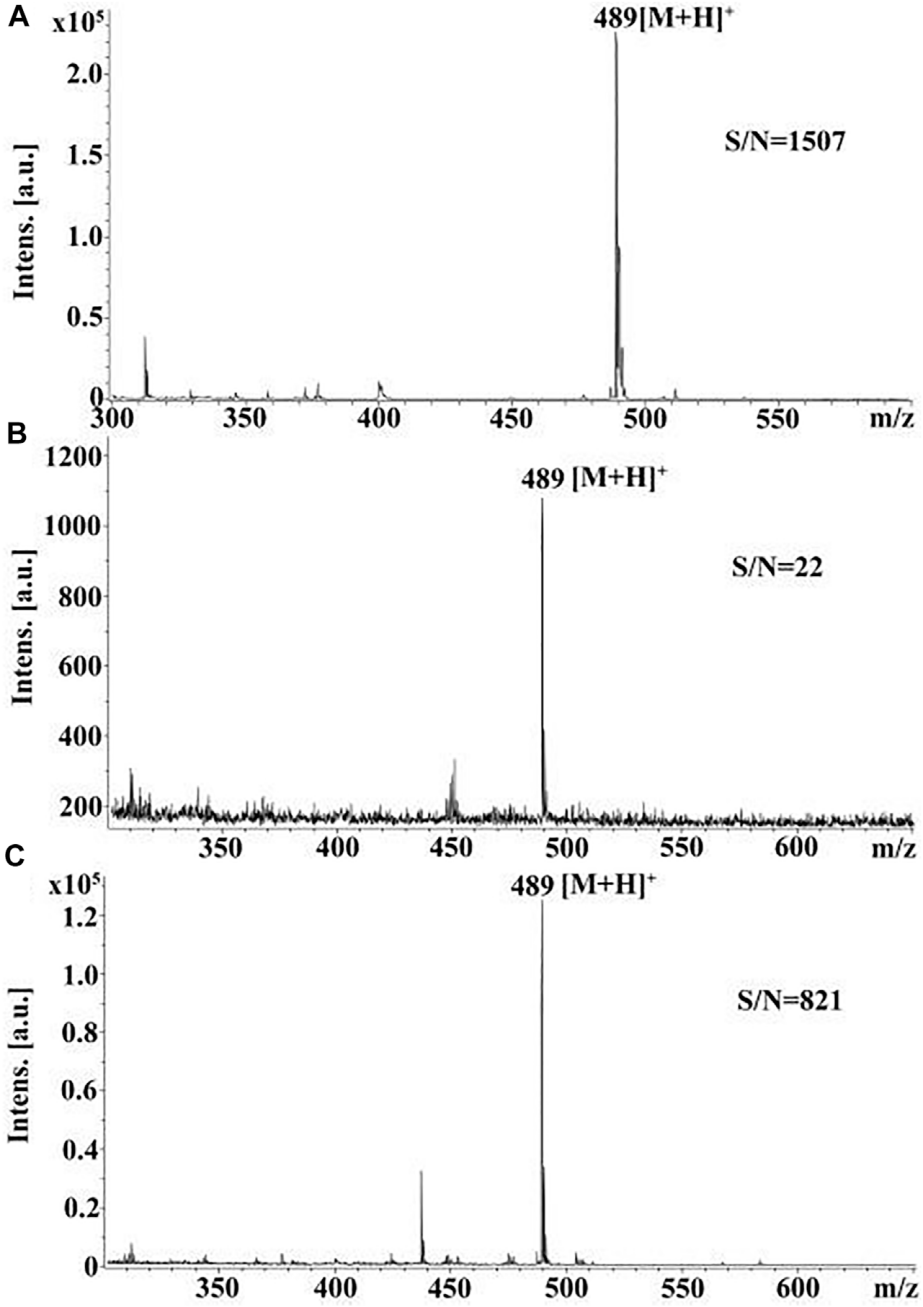
MALDI mass spectra of vardenafil registered from **(A)** steel MALDI target using AT as the matrix, **(B)** TLC plate after elution and using AT as the matrix, and **(C)** TLC plate after elution using the composite matrix (AT-glycerol-graphite). Figure reprinted with permission from Esparza et al. (2016). Copyright (2016) Elsevier B. V.

Composite glycerol/graphite/aromatic acid matrices were also proposed to avoid spot blurring and enhance desorption/ionization of the analytes (Esparza et al., 2016). Mixtures of traditional matrixes and graphite were dispersed in glycerin and applied on spots using a brush. The authors supposed that glycerol allows preventing secondary chromatographic processes and increasing analyte concentration on the surface of TLC plates. These factors increased the signals of analytes and reproducibility of mass spectra (Figure 3).

Dopants also sometimes play a very important role in ionization processes (Ali et al., 2017). In case of the previously mentioned GALDI-MS, sodium and potassium cations found in pencil lead take part in the formation of adducts of molecules. But, Y. Dong et al. found out that the concentration of alkali ions causing the formation of the corresponding adduct is higher on the surface of TLC plates (Dong et al., 2017). So, if the sorbent layer is scratched, the intensities of its peaks decrease, reducing the reproducibility of the results.

### Derivatization

**FIGURE 4:**
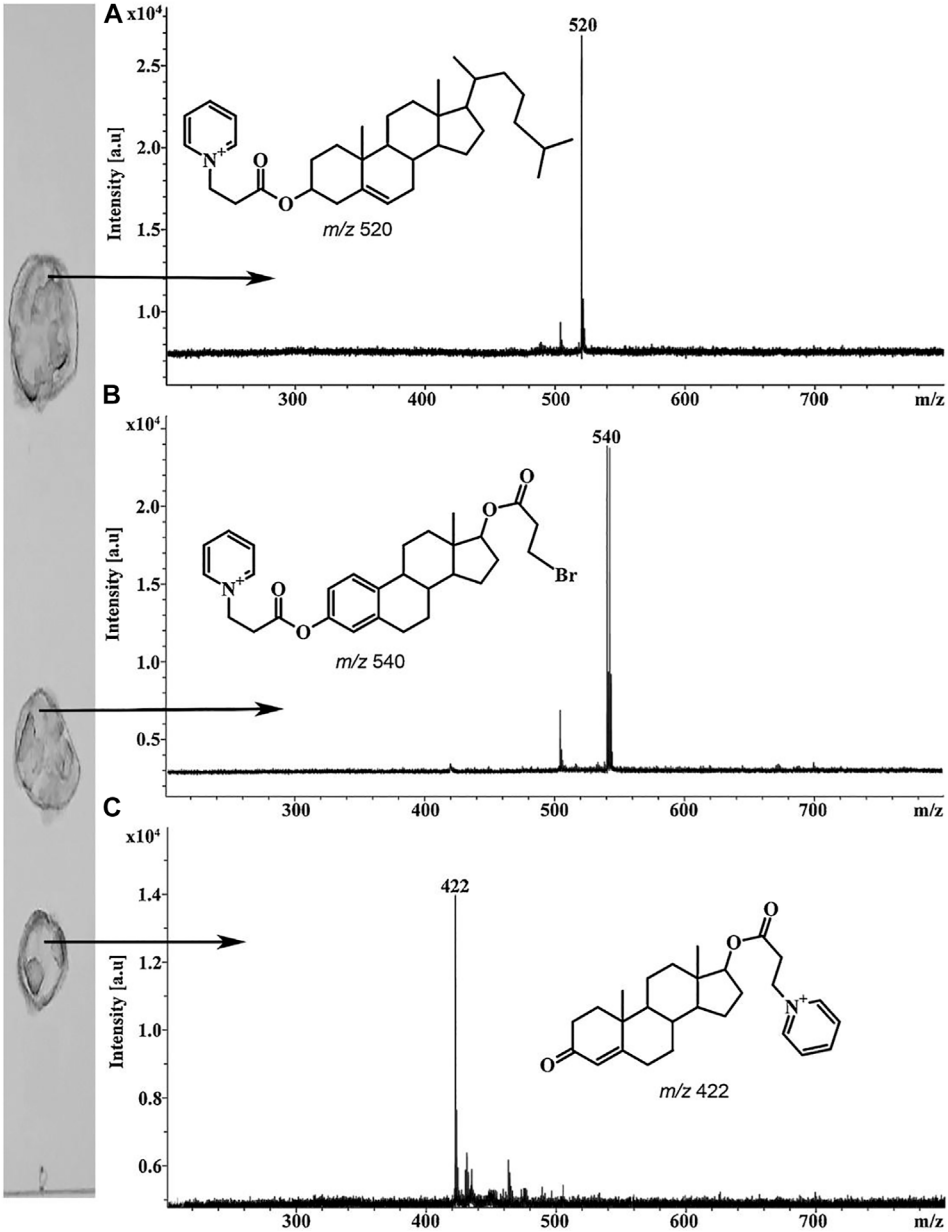
MALDI mass spectra of steroid alcohols derivatized on the TLC plate cholesterol **(A)**, β-estradiol **(B)**, positions of bromopropionyl and pyridiniumpropionyl groups may vary and testosterone **(C)**. Figure reprinted with permission from Esparza et al. (2018). Copyright (2018) Elsevier B. V.

Planar chromatography is one of the first analytical methods where derivatization procedures were applied mainly for spot visualization (Zarzycki, 2015). Derivatization is also widely used in MALDI-MS and MALDI imaging for enhancing desorption/ionization efficiencies of the analytes (Zaikin and Halket, 2009; Zaikin and Borisov, 2021). However, chemical modification procedures are much less popular in case of TLC/MALDI. Nevertheless, such approaches look rather promising for the analysis of non- or low-polar analytes by this method. Thus, for example, a previously developed procedure for fixed-charge derivatization of alcohols (Borisov et al., 2014) was used for TLC/MALDI (Esparza et al., 2018). The derivatization agents, including 3-bromopropionyl chloride and pyridine, were applied directly on the elution zones of the analytes on the developed TLC plates. MALDI mass spectra registered from the plates contained intense peaks of the corresponding derivatives (Figure 4).

**FIGURE 5:**
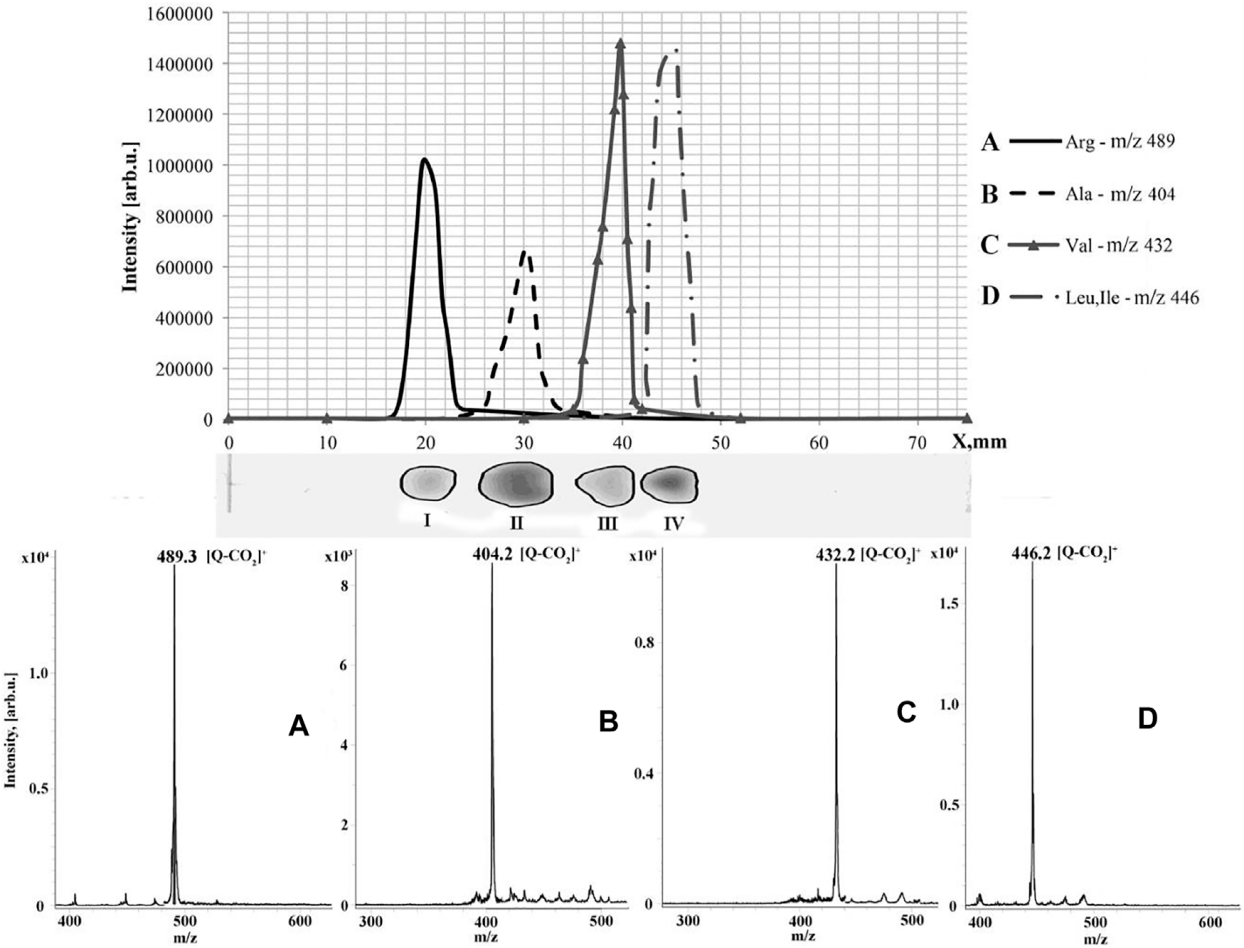
Graphic and real thin-layer chromatograms (top two images) of dietary supplement and recorded MALDI mass spectra of TDPMH derivatives of the detected amino acids (bottom images). Figure reprinted with permission from Esparza et al. (2020). Copyright (2020) Elsevier B. V.

A similar approach was used for TLC/MALDI analysis of primary amines (Borisov et al., 2019). In this case, tris(2,6-dimethoxyphenyl)methilium hexafluorophosphate was used for the derivatization to yield cyclic acridine–like fixed-charge derivatives. The modification of analytes also causes change in the color of the spots. The latter is very useful to avoid using additional procedures to determine analyte elusion zones. The same derivatization agent was involved in the development of a method for analysis of aminoacids (Esparza et al., 2020). The derivatives of α-aminoacids underwent elimination of carbon dioxide under laser irradiation, but the formed ions produced intense peaks in the registered MALDI mass spectra. The proposed approach was successfully applied for the analysis of dietary supplements (Figure 5).

### Applications

The unique opportunities of the TLC/MALDI systems result in wide utilization of this method in the analytics of complex organic mixtures. One of the areas of interest in current analytical chemistry is lipid analysis. Cebolla et al. (2021) have recently presented a review on HPTLC contribution to lipid analysis in complex matrices during the period 2010–2021, when hyphenation of TLC to MS methods had experienced significant growth. Authors demonstrated that HPTLC separation of lipids on classes allowed the subsequent analysis by MALDI-TOF, resolving the difficulties in the analysis of acidic glycerophospholipid species due to ion suppression by phosphatidylcholine species. MALDI also allows structural identification of untargeted lipids which are ionized from the surface by continuous scanning along the plate track. In a similar way in which MS imaging allows the recording of mass spectra from the slices of biological tissues, MALDI may be used for screening the distribution of the lipids and other organic compounds on the HPTLC plate. Although most HPTLC-MALDI studies are performed with UV lasers using a matrix, other alternatives are also presented in the literature. In the study by Taki (2015), glycosphingolipids were separated on the HPTLC plate and then transferred to a plastic membrane which was fixed on a MALDI adapter (blotting process). Direct HPTLC/LDI without the matrix was realized using an IR laser by Leopold et al. (2018). This method allowed ablating more material per laser pulse on the order of a few micrometers in depth regarding UV lasers, improving sensitivity of the process, which is important taking into consideration the TLC plate thickness.

Ferey et al. (2017) have reported the utilization of TLC/MALDI-TOF-MS for the screening of invertase substrates in complex matrices. BfrA, a specific β-D-fructofuranosidase from *Leishmania major*, was chosen by the authors as a model enzyme to screen biological activity in plant extracts due to its capacity to hydrolyze specific carbohydrates. The first part of the approach was in differential analysis by TLC densitometry to determine the zones in the plant extract between the blank and enzymatic reaction. Zones of interest were then subsequently investigated *via* TLC/MALDI-TOF-MS for the identification of bioactive molecules. The development of the method demanded the solution of different analytical problems, such as separation of isomers (glucose and fructose), elimination of the high-matrix effect, and the analysis of polar molecules with low molar masses (sugars), which is a challenging problem itself while interacting about MALDI. However, the method occurred to be feasible for the analysis of bioactive molecules in complex mixtures containing interfering compounds (e.g., proteins and salts).

TLC/MALDI is widely used for the analysis of lipids (Engel and Schiller, 2021). Thus, Kouzel et al., (2017 have investigated the TLC-IR-MALDI-MS as an analytical tool for the detection and structural characterization of glyco and phospholipids directly from the TLC plate. The authors have coupled a pulsed IR-MALDI laser to a hybrid Synapt G2-S mass spectrometer and used the suggested ion source configuration for TLC-IR-MALDI-MS imaging of neutral glycosphingolipids, obtained from human colon epithelial HCT-8 cells. The aim of the article was the detection of the two glycosphingolipids, namely, globotriaosylceramide and globotetraosylceramide, which are the main receptors for Shiga toxins produced by enterohemorrhagic *Escherichia coli*. The direct TLC-IR-MALDI-MS analysis allowed the successful visualization of the chromatographic separation of the various lipo forms of globotriaosylceramide and globotetraosylceramide. The developed method allowed the fast and reliable overview of the glycosphingolipid composition of the investigated cell line of high medical relevance. This possibility may also be useful in glycolipidomic studies of complex biological matrices; for example, for globotriaosylceramide imaging, Kouzel et al. (2017) detected a higher extent of heterogeneity in lipo forms than that up to this point, analyzing monohexosyl and dihexosylceramide species. According to the structures of the biosynthesis precursor glycosphingolipids, namely, glucosylceramide and lactosylceramide, globotriaosylceramide species with sphingosine and fatty acid variations were identified by authors at m/z 1158.78 (d18:1, C24:0), m/z 1156.74 (d18:1, C24:1), m/z 1130.74 (d18:1, C22:0), and m/z 1046.66 (d18:1, C16:0). It is worth noting that the C24:0 fatty acid- and C24:1 fatty acid-carrying globotriaosylceramide species, differing only in the double bond in the acyl chain, were not separated on the chromatographic step due to their identical chromatographic properties in the utilized conditions. However, these compounds together formed a chromatographic zone which was definitely separated from the spots on the TLC plate containing globotriaosylceramide with C22:0 and C16:0 acyls. The additional MS data allowed to suggest that the colon epithelial cells synthesize globotriaosylceramide species with dihydroxylated sphinganine (d18:0), detected at m/z 1048.66 (d18:0, C16:0), and trihydroxylated sphinganine (t18:0) at m/z 1064.66 (t18:0, C16:0).

Torretta et al. (2016) have used HPTLC-MALDI-MS for the investigation of sphingolipid and glycosphingolipid profiles in the muscle, brain, and serum for creating a database of molecules for preclinical and clinical investigations. Based on the properties of the studied tissues and fluids, the specific protocols for lipid extraction were used by the authors to maximize the HPTLC-MALDI-MS analytical throughput both for lipids extracted in the organic and aqueous phases. The received result allowed the authors to develop the database of specie-specific molecules, which may contribute to preclinical and clinical studies. The performed study indicated that alkaline hydrolysis was necessary for the detection of low-abundant species in serum and muscle tissues. The high hydrophobicity of ceramide was overcome by the development of the HPTLC plate in a specific eluent [chloroform/methanol 50:3.5 (v/v)], resulting in increased number and intensity of low-abundant ceramide species.

Kroslakova et al. (2016) have reported on the direct coupling of HPTLC with MALDI-TOF-MS for qualitative detection of flavonoids on phytochemical fingerprints. It is known that TLC fingerprints of plant raw materials and extracts for various applications usually focus on phenol acids and flavonoids. The TLC/MALDI-TOF-MS method has been applied by the authors for the development of fingerprints of flavonoids. The authors have demonstrated the feasibility of direct coupling of HPTLC with UV-MALDI-TOF-MS for the determination of the molecular mass of the flavonol glycoside, that is, rutin, and flavone glycoside, that is, luteolin-7-O-glucoside, and the corresponding aglycones, that is, quercetin and luteolin. After the primary TLC separation on the MS-grade plates, the developed chromatogram was treated with 2,5-dihydroxybenzoic acid as a MALDI matrix, dried, and scanned by UV-MALDI-TOF-MS. All the studied compounds were detected in MALDI-TOF mass spectra. This is particularly important for the coeluted compounds—aglycones luteolin and quercetin, which could not have been distinguished by the densitometric HPTLC method. The authors have demonstrated the potential of MALDI-TOF-MS for the analysis of low molar mass fingerprints of flavonoids directly from their HPTLC chromatogram.

## Thin-Layer Chromatography and Novel Desorption/Ionization Mass-Spectrometry Techniques

Though MALDI-MS is the most used approach for direct detection of analytes from TLC plates, there also several desorption/ionization methods which are also capable of such analyses. Thus, even in the 1980s, it was shown that fast atom bombardment (Chang et al., 1984) and secondary ion mass spectrometry (Kushi and Handa, 1985) can be conjugated with planar chromatography. The development of ambient ionization methods, such as “direct analysis in real-time” (DART) and desorption electrospray ionization (DESI), makes it possible to ionize molecules from TLC plates with minimum sample preparation (Morlock and Schwack, 2010). On the one hand, such approaches look more promising than TLC/MALDI because they do not require any matrix, making the developed methods more reproducible and easy to use. On the other hand, laser-based vacuum systems such as MALDI guarantee high spatial resolution and minimum side-ionization processes.

The first study demonstrating the power of the combination of TLC and ambient ionization MS was published shortly after the presentation of DESI (Van Berkel et al., 2005). The method is based on spraying of the TLC surface with ultrasmall solvent droplets at velocities higher than 100 m/s, causing desorption of the ionized molecules of the analytes. The latter are transferred to the interface of the MS and detected (Manikandan, et al., 2016). The main drawback of this method is the dependence of the size of desorption spots on TLC plates on solvent flow rates (Bagatela, et al., 2015). Low flow rates lead to decreasing desorption of analytes and low intensities of corresponding ions, whereas high flow rates produce large spots causing diffusion of analytes across TLC plates, decreasing spatial resolution, and co-ionization of compounds with close R_f_. Nevertheless, the approach was used, for example, to develop methods for detecting components of thermochromic inks for forensic purposes (Khatami et al., 2017). A very interesting combination of TLC/DESI with ion mobility spectroscopy/mass spectrometry (IMS/MS) was described by Claude et al. (2020). In this case, IMS was necessary to resolve co-eluting isomers of ecdysteroids, but, in fact, the proposed multidimensional separation system can be used for the analysis of very complex mixtures of various origins.

DART mass spectrometry operates using Penning ionization principles: excited atoms of gas (mainly helium) interact with molecules of ambient air components producing secondary ions, which desorb/ionize compounds from the surface of the analyzed object (Rondeau, 2017). This ionization mechanism is more preferable for TLC than DESI because using gas streams avoids diffusion of analytes (Morlock and Ueda, 2007). Moreover, rather high temperature of gas enhances desorption of analytes from plates, although some thermal decompositions of labile compounds can occur. The method can also be combined with online derivatization to enhance the ionization efficiencies of the analytes (Borisov et al., 2019).

**FIGURE 6:**
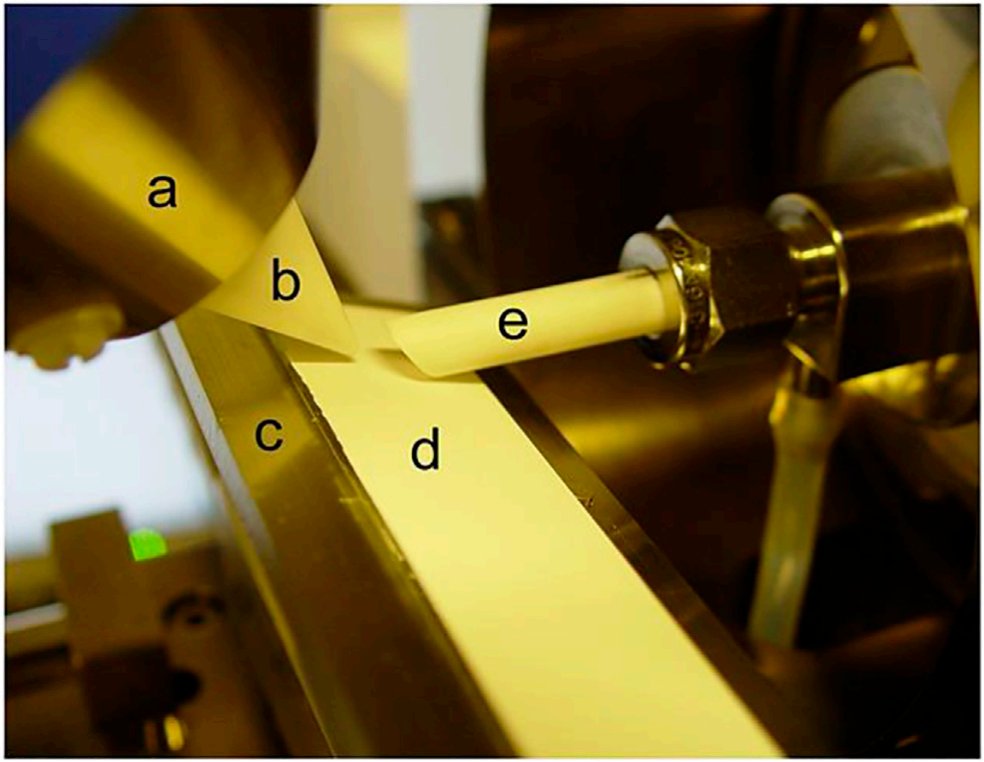
DART ion source with possibility to change the gas flow angle for desorption/ionization of analytes from TLC plates. Reprinted with permission from Häbe and Morlock (2015). Copyright © 2015 John Wiley & Sons, Ltd.

The first version of the commercial DART ion source was inconvenient for TLC analysis because the gas stream was directed to the orifice of the MS interface and the analysis required cutting of the plates. Though a special adapter increases the efficiency of desorption/ionization and reproducibility of the results was proposed by Ovcharov et al. (2017), the problem was completely overcome with the release of new generation of DART ion sources, allowing to change the gas flow angle (so-called reflection scanning) and having TLC holders (Figure 6). The sensitivity of TLC/DART can also be increased using shortened source caps (Häbe and Morlock, 2015). There are also approaches allowing the visualization of the gas impact region on plates using neon additives to helium (Chernetsova and Morlock, 2015) or substances changing their color upon heating (Chernetsova et al., 2011).

All abovementioned improvements have rapidly increased the popularity of TLC/DART, and now, it is the most common combination of ambient mass spectrometry with planar chromatography. Thus, this approach is often used for the characterization of plant extracts. For example, DART-MS coupled with the ion trap mass analyzer was used for express identification of alkaloids extracted from plants and separated by TLC (Chen, et al., 2018). The combination of TLC with derivatization, bioautography, and DART-MS was successfully applied by Bañuelos-Hernández et al. (2020) for the analysis of Mexican *Plectranthus amboinicus* (Lour.) essential oil. Comparison of HPTLC-UV/Vis chromatograms before and after derivatization using the anisaldehyde–sulfuric acid reagent and HPTLC-Vis-EDA autograms allowed determining bioactive compound zones, which then were subjected to analysis by DART with a high-resolution mass analyzer. A similar approach was used for bioanalytical profiling of sunflower leaves (Móricz et al., 2018).

TLC/DART analysis can also be used for quantitative analysis of various compounds. Eichner and Spangenberg (2019) have developed a method for separation and quantitative determination of caffeine, artemisinin, and equol. The latter was also an object of interest in the study by Peters and Spangenberg, (2019). The comparison of results achieved using TLC-UV and TLC/DART for the quantification of this substance in cattle manure extracts clearly shows that selectivity of mass spectrometry allows obtaining more exact results. The authors underline that in case of UV detection, corresponding peaks were significantly broader, causing more errors.

A very promising approach was developed by Chen et al. (2021). In this study, DART-MS was combined with a laser system, in which irradiation significantly increased desorption of analytes. A similar method has already been published, but the authors used an expensive multi-wavelength laser device (Zhang et al., 2012), whereas the mentioned approach is based on a low-cost and easy-to-install laser system. It also allowed decreasing the temperature of the gas stream, which is rather high for the TLC/DART experiment as usual. The proposed approach was validated using various synthetic compounds and applied for the analysis of natural herbal medicines.

There are also a significant number of other home-built systems allowing desorption/ionization of analytes from TLC plates. Their descriptions are summarized in Table 1. Although such systems are not commercially produced, they demonstrate great potential for further development and may take an important place in TLC/MS analysis. Laser-based systems appear to be most promising because they allow achieving the highest spatial resolution, which is very important for the analysis of co-eluted analytes.

**TABLE 1:** Home-built interfaces for TLC/MS systems.

Desorption/ionization principle	Objects/analytes	Results	References
Laser desorption–low-temperature plasma (LD-LTP)	Tea, coffee beans, and soluble coffee extracts	Quantitative analysis of compounds in complex matrices, possibility of a low-cost laser system used in combination with an activated carbon matrix	Garcia-Rojas et al. (2020)
Low-temperature plasma (LTP)	Pharmaceuticals and biologically active compounds	Desorption/ionization efficiencies depend on the nature of solvents and analytes; LODs are compared to other approaches	Gong et al. (2020)
Laser desorption– low-temperature plasma (LD-LTP)	Pharmaceuticals and biologically active compounds	High spatial resolution and possibility to decrease LODs by increasing laser spot size	Gong et al. (2020)
Desorption atmospheric pressure photoionization (DAPPI)	Human lipids and plant oils	Detection of fatty acid diols and glycerol esters, cholesterol and its derivatives, and squalene	Rejsek et al. (2016)
Desorption atmospheric pressure chemical ionization (DAPCI)	Amino acids and drugs	Detection of amino acid and pharmaceutical compounds, linear signal	Winter et al. (2015)
Desorption atmospheric pressure chemical ionization (DAPCI)	Hop acids	Semi-quantitative determination of α- and β-acid ratio	Winter et al. (2017)
Sawtooth TLC-ESI/MS	Dyes	Detection of dyes using a very simple experimental design	Cheng et al. (2019)
Flowing atmospheric pressure afterglow (FAPA)	Pyrazole derivatives, alkaloids, steroids, and drugs	LODs comparable with other ambient methods achieved using a simple laser pointer for ablation, quantitative results	Ceglowski et al. (2015)
			Kuhlmann et al. (2019)
Electrostatic spray ionization (ESTASI)	Dyes and drugs	Detection of all tested compounds, low LODs	Zhong et al. (2015)
Electrostatic field–induced spray ionization (EFISI)	Herbal extracts	Detection of alkaloids, flavonoids, phenoic acids, lignans, coumarins, anthraquinones, monoterpenoids, sesquiterpenoids, diterpenoids, and triterpenoids	Zhang et al. (2019)
			Zhang et al. (2020)
Desorption/ionization induced by neutral clusters (DINeC)	Oligopeptides and extracts from yolk of a chicken egg	Extremely soft ionization technique allowing desorption ionization without any fragmentation	Heep et al. (2019)
Diode laser thermal vaporization–inductively coupled plasma (DLTV-ICP)	Algae	Reproducible quantification of selenium in algae using a low-cost experiment design; the results are comparable to HPLC-ICP MS	Bednarik et al. (2018)

## Conclusion

Reproducibility and high spatial resolution achieved by MALDI-MS are still keeping this method as the most widely used desorption/ionization technique for the detection of analytes from TLC plates. Further developments of this method, including new chromatographic sorbents and matrix compounds/mixtures, and novel sample preparation procedures guarantee its popularity for the analysis of various objects. Most notably, there are new studies introducing TLC/MALDI as a useful tool in new research fields such as, for example, petroleomics. However, the fast growing number of ambient ionization techniques offers a good alternative for MALDI. These methods allow developing cost-effective, robust, and express approaches for the detection of various classes of analytes from TLC plates. We believe that all these MS ionization approaches will keep one of the first chromatographic separation methods actual for a lot of purposes.

## References

[B1] Ali A. Shahid N. Musharraf S. G. (2017). Application of Dyes as Doping Agents in MALDI-MS Matrices for the Signal Enhancement of Proteins. RSC Adv. 7, 6598–6604. 10.1039/C6RA27156A

[B2] Bagatela B. S. Lopes A. P. Cabral E. C. Perazzo F. F. Ifa D. R. (2015). High-performance Thin-Layer Chromatography/desorption Electrospray Ionization Mass Spectrometry Imaging of the Crude Extract from the Peels of Citrus Aurantium L. (Rutaceae ). Rapid Commun. Mass. Spectrom. 29, 1530–1534. 10.1002/rcm.7246 26212168

[B3] Bañuelos-Hernández A. E. Azadniya E. Ramírez Moreno E. Morlock G. E. (2020). Bioprofiling of Mexican Plectranthus Amboinicus (Lour.) Essential Oil via Planar Chromatography-Effect-Directed Analysis Combined with Direct Analysis in Real Time High-Resolution Mass Spectrometry. J. Liquid Chromatogr. Relat. Tech. 43, 344–350. 10.1080/10826076.2020.1737542

[B4] Barker J. Ramotowski R. Nwokoye J. (2016). The Effect of Solvent Grade on Thin Layer Chromatographic Analysis of Writing Inks. Forensic Sci. Int. 266, 139–147. 10.1016/j.forsciint.2016.05.003 27262685

[B5] Beate Fuchs K. M. Fuchs B. Lemmnitzer K. Süß R. Griesinger H. Minarik S. (2015). Combining TLC Separation with MS Detection - A Revival of TLC. J. Glycomics Lipidomics 05, 1–3. 10.4172/2153-0637.1000e125

[B6] Bednařík A. Kuta J. Vu D. L. Ranglová K. Hrouzek P. Kanický V. (2018). Thin-layer Chromatography Combined with Diode Laser thermal Vaporization Inductively Coupled Plasma Mass Spectrometry for the Determination of Selenomethionine and Selenocysteine in Algae and Yeast. J. Chromatogr. A 1533, 199–207. 10.1016/j.chroma.2017.12.017 29248347

[B7] Borisov R. Esparza C. Polovkov N. Topolyan A. Zaikin V. (2019). An Approach to Analysis of Primary Amines by a Combination of Thin‐layer Chromatography and Matrix‐assisted Laser Desorption Ionization Mass Spectrometry in Conjunction with post‐chromatographic Derivatization. J. Sep. Sci. 42, 3470–3478. 10.1002/jssc.201900644 31515926

[B8] Borisov R. S. Esparza C. Goriainov S. V. Zaikin V. G. (2019). Suitable *In-Situ* Derivatization of Alcohols by Reaction with Basic Amines in Direct Analysis in Real Time Mass Spectrometry. Talanta 200, 31–40. 10.1016/j.talanta.2019.03.037 31036190

[B9] Borisov R. S. Polovkov N. Y. Zhilyaev D. I. Esparza C. A. Zaikin V. G. (2014). Combination of Graphite-Assisted Laser Desorption/ionization (GALDI) Mass Spectrometry with Thin Layer Chromatography. J. Anal. Chem. 69, 1351–1355. 10.1134/S1061934814140032

[B10] Borisov R. S. Zhilyaev D. I. Polovkov N. Y. Zaikin V. G. (2014). Simple Approach to Derivatization of Alcohols and Phenols for the Analysis by Matrix(surface)-Assisted Laser Desorption/ionization Time-Of-Flight Mass Spectrometry. Rapid Commun. Mass. Spectrom. 28, 2231–2236. 10.1002/rcm.7008 25279736

[B11] Cagniant D. Nosyrev I. Cebolla V. Vela J. Membrado L. Gruber R. (2001). Structural Modifications of Petroleum Asphaltenes by Reductive Alkylation Investigated by TLC-FID. Fuel 80, 107–115. 10.1016/S0016-2361(00)00041-7

[B12] Calvano C. D. Monopoli A. Cataldi T. R. I. Palmisano F. (2018). MALDI Matrices for Low Molecular Weight Compounds: an Endless story? Anal. Bioanal. Chem. 410, 4015–4038. 10.1007/s00216-018-1014-x 29682685

[B13] Cebolla V. L. Jarne C. Vela J. Garriga R. Membrado L. Galbán J. (2021). Scanning Densitometry and Mass Spectrometry for HPTLC Analysis of Lipids: The Last 10 Years. J. Liquid Chromatogr. Relat. Tech. 44, 148–170. 10.1080/10826076.2020.1866600

[B14] Cebolla V. L. Lázaro M. J. Herod A. A. (2016). Petroleum Products-Thin Layer (Planar) Chromatography☆. Ref. Module Chem. Mol. Sci.Chem. Eng. 25, 3690–3701. 10.1016/B978-0-12-409547-2.12669-1

[B15] Cegłowski M. Smoluch M. Reszke E. Silberring J. Schroeder G. (2015). Flowing Atmospheric Pressure Afterglow Combined with Laser Ablation for Direct Analysis of Compounds Separated by Thin-Layer Chromatography. Anal. Bioanal. Chem. 408, 815–823. 10.1007/s00216-015-9165-5 26563110 PMC4709388

[B16] Chang T. T. Lay J. O. Francel R. J. (1984). Direct Analysis of Thin-Layer Chromatography Spots by Fast Atom Bombardment Mass Spectrometry. Anal. Chem. 56, 109–111. 10.1021/ac00265a030

[B17] Chen Y. Li L. Xu R. Li F. Gu L. Liu H. (2021). Characterization of Natural Herbal Medicines by Thin-Layer Chromatography Combined with Laser Ablation-Assisted Direct Analysis in Real-Time Mass Spectrometry. J. Chromatogr. A 1654, 462461. 10.1016/j.chroma.2021.462461 34438305

[B18] Chen Z. Wang M. Yang Y. Du X. Zhang Z. Li Y. (2018). Qualitative and Quantitative Analysis of Porana Sinensis Hemsl by UHPLC‐Q‐Exactive MS, TLC Autographic Method and DART‐MS. Phytochem. Anal. 30, 311–319. 10.1002/pca.2814 30569488

[B19] Cheng S.-C. Bhat S. M. Lee C.-W. Shiea J. (2019). Simple Interface for Scanning Chemical Compounds on Developed Thin Layer Chromatography Plates Using Electrospray Ionization Mass Spectrometry. Analytica Chim. Acta 1049, 1–9. 10.1016/j.aca.2018.10.042 30612639

[B20] Chernetsova E. S. Morlock G. E. (2015). Aspects of Surface Scanning by Direct Analysis in Real Time Mass Spectrometry Employing Plasma Glow Visualization. Rapid Commun. Mass. Spectrom. 29, 1242–1252. 10.1002/rcm.7221 26395608

[B21] Chernetsova E. S. Revelsky A. I. Morlock G. E. (2011). Some New Features of Direct Analysis in Real Time Mass Spectrometry Utilizing the Desorption at an Angle Option. Rapid Commun. Mass. Spectrom. 25, 2275–2282. 10.1002/rcm.5112 21766371

[B22] Claude E. Tower M. Lafont R. Wilson I. D. Plumb R. S. (2020). High Performance Thin-Layer Chromatography of Plant Ecdysteroids Coupled with Desorption Electrospray Ionisation-Ion Mobility-Time of Flight High Resolution Mass Spectrometry (HPTLC/DESI/IM/ToFMS). Chromatographia 83, 1029–1035. 10.1007/s10337-020-03917-9

[B23] Dong Y. Ferrazza R. Anesi A. Guella G. Franceschi P. (2017). TLC Surface Integrity Affects the Detection of Alkali Adduct Ions in TLC-MALDI Analysis. Anal. Bioanal. Chem. 409, 5661–5666. 10.1007/s00216-017-0501-9 28730308

[B24] Eichner F. Spangenberg B. (2019). Optimized Determination of Caffeine, Equol, and Artemisinin by High-Performance Thin-Layer Chromatography-Direct Analysis in Real Time-Time of Flight-Mass Spectrometry. JPC - J. Planar Chromatogr. - Mod. TLC 32, 197–203. 10.1556/1006.2019.32.3.4

[B25] Engel K. M. Schiller J. (2021). The Value of Coupling Thin-Layer Chromatography to Mass Spectrometry in Lipid Research - a Review. J. Chromatogr. B 1185, 123001. 10.1016/j.jchromb.2021.123001 34715571

[B26] Esparza C. Borisov R. S. Polovkov N. Y. Zaikin V. G. (2018). Post-chromatographic Fixed-Charge Derivatization for the Analysis of Hydroxyl-Containing Compounds by a Combination of Thin-Layer Chromatography and Matrix-Assisted Laser Desorption/ionization Mass Spectrometry. J. Chromatogr. A 1560, 97–103. 10.1016/j.chroma.2018.05.025 29803430

[B27] Esparza C. Borisov R. S. Varlamov A. V. Zaikin V. G. (2016). Composite Glycerol/graphite/aromatic Acid Matrices for Thin-Layer Chromatography/matrix-Assisted Laser Desorption/ionization Mass Spectrometry of Heterocyclic Compounds. J. Chromatogr. A 1470, 118–122. 10.1016/j.chroma.2016.09.075 27720171

[B28] Esparza C. Polovkov N. Y. Topolyan A. P. Borisov R. S. Zaikin V. G. (2020). Suitable Analysis of α-amino Acids by a Direct Combination of Thin-Layer Chromatography and Matrix-Assisted Laser Desorption/ionization Mass Spectrometry in Conjunction with post-chromatographic Fixed-Charge Derivatization. J. Chromatogr. A 1626, 461335. 10.1016/j.chroma.2020.461335 32797820

[B29] Ferey J. Da Silva D. Lafite P. Daniellou R. Maunit B. (2017). TLC-UV Hyphenated with MALDI-TOFMS for the Screening of Invertase Substrates in Plant Extracts. Talanta 170, 419–424. 10.1016/j.talanta.2017.04.040 28501191

[B30] Fougère L. Da Silva D. Destandau E. Elfakir C. (2018). TLC-MALDI-TOF-MS-based Identification of Flavonoid Compounds Using an Inorganic Matrix. Phytochem. Anal. 30, 218–225. 10.1002/pca.2807 30474345

[B31] García-Rojas N. S. Moreno-Pedraza A. Rosas-Román I. Ramírez-Chávez E. Molina-Torres J. Winkler R. (2020). Mass Spectrometry Imaging of Thin-Layer Chromatography Plates Using Laser Desorption/low-Temperature Plasma Ionisation. Analyst 145, 3885–3891. 10.1039/D0AN00446D 32297600

[B32] Gong X. Zhang D. Embile I. B. She Y. Shi S. Gamez G. (2020). Low-Temperature Plasma Probe Mass Spectrometry for Analytes Separated on Thin-Layer Chromatography Plates: Direct vs Laser Assisted Desorption. J. Am. Soc. Mass. Spectrom. 31, 1981–1993. 10.1021/jasms.0c00246 32810399

[B33] Griesinger H. Fuchs B. Süß R. Matheis K. Schulz M. Schiller J. (2014). Stationary Phase Thickness Determines the Quality of Thin-Layer Chromatography/matrix-Assisted Laser Desorption and Ionization Mass Spectra of Lipids. Anal. Biochem. 451, 45–47. 10.1016/j.ab.2014.02.002 24530848

[B34] Häbe T. T. Morlock G. E. (2015). Improved Desorption/ionization and Ion Transmission in Surface Scanning by Direct Analysis in Real Time Mass Spectrometry. Rapid Commun. Mass. Spectrom. 30, 321–332. 10.1002/rcm.7434 26689161

[B35] Häbe T. T. Morlock G. E. (2015). Quantitative Surface Scanning by Direct Analysis in Real Time Mass Spectrometry. Rapid Commun. Mass. Spectrom. 29, 474–484. 10.1002/rcm.7127 26160413

[B36] Heep J. Tuchecker P. H. K. Gebhardt C. R. Dürr M. (2019). Combination of Thin-Layer Chromatography and Mass Spectrometry Using Cluster-Induced Desorption/Ionization. ACS Omega 4, 22426–22430. 10.1021/acsomega.9b03060 31909324 PMC6941192

[B37] Hillenkamp F. Peter-Katalinic J. (2013). MALDI MS: A Practical Guide to Instrumentation, Methods and Applications. Second Edition. Weinheim: Wiley‐VCH Verlag GmbH & Co. KGaA. 10.1002/9783527335961

[B38] Hu B. Xin G.-z. So P.-K. Yao Z.-P. (2015). Thin Layer Chromatography Coupled with Electrospray Ionization Mass Spectrometry for Direct Analysis of Raw Samples. J. Chromatogr. A 1415, 155–160. 10.1016/j.chroma.2015.08.055 26362806

[B39] Hynstova V. Sterbova D. Klejdus B. Hedbavny J. Huska D. Adam V. (2018). Separation, Identification and Quantification of Carotenoids and Chlorophylls in Dietary Supplements Containing Chlorella Vulgaris and Spirulina Platensis Using High Performance Thin Layer Chromatography. J. Pharm. Biomed. Anal. 148, 108–118. 10.1016/j.jpba.2017.09.018 28987995

[B40] Khatami A. Prova S. S. Bagga A. K. Yan Chi Ting M. Brar G. Ifa D. R. (2017). Detection and Imaging of Thermochromic Ink Compounds in Erasable Pens Using Desorption Electrospray Ionization Mass Spectrometry. Rapid Commun. Mass. Spectrom. 31, 983–990. 10.1002/rcm.7867 28370721

[B41] Kim H.-H. Han S.-P. Kim J.-K. Kim Y.-J. (2011). Detection of Long Alkyl Esters of Succinic and Maleic Acid Using TLC-MALDI-MS. Bull. Korean Chem. Soc. 32, 915–920. 10.5012/BKCS.2011.32.3.915

[B42] Kobylis P. Stepnowski P. Caban M. (2021). Review of the Applicability of Ionic Liquid Matrices for the Quantification of Small Molecules by MALDI MS. Microchemical J. 164, 105983. 10.1016/j.microc.2021.105983

[B43] Kouzel I. U. Soltwisch J. Pohlentz G. Schmitz J. S. Karch H. Dreisewerd K. (2017). Infrared MALDI Mass Spectrometry Imaging of TLC-Separated Glycosphingolipids with Emphasis on Shiga Toxin Receptors Isolated from Human colon Epithelial Cells. Int. J. Mass Spectrom. 416, 53–60. 10.1016/j.ijms.2016.11.008

[B44] Kroslakova I. Pedrussio S. Wolfram E. (2016). Direct Coupling of HPTLC with MALDI-TOF MS for Qualitative Detection of Flavonoids on Phytochemical Fingerprints. Phytochem. Anal. 27, 222–228. 10.1002/pca.2621 27313160

[B45] Kucherenko E. Kanateva A. Kurganov A. Borisov R. Pirogov A. (2018). Monolithic Thin‐layer Chromatography Plates with Covalently Bonded Matrix for Hyphenation with Matrix‐assisted Laser Desorption/ionization. J. Sep. Sci. 41, 4387–4393. 10.1002/jssc.201800679 30281906

[B46] Kucherenko E. Kanateva A. Pirogov A. Kurganov A. (2019). Recent Advances in the Preparation of Adsorbent Layers for Thin-Layer Chromatography Combined with Matrix-Assisted Laser Desorption/ionization Mass-Spectrometric Detection. J. Sep. Sci. 42, 415–430. 10.1002/jssc.201800625 30156034

[B47] Kuhlmann C. Heide M. Engelhard C. (2019). Fast Screening and Quantitative Mass Spectral Imaging of Thin-Layer Chromatography Plates with Flowing Atmospheric-Pressure Afterglow High-Resolution Mass Spectrometry. Anal. Bioanal. Chem. 411, 6213–6225. 10.1007/s00216-019-02013-8 31317240

[B48] Kumar M. Kuzhiumparambil U. Ralph P. J. Contreras-Porcia L. (2017). “Polyamines,” in Algal Green Chemistry. Editors Rastogi R. Madamwar D. Pandey A. (Amsterdam: Elsevier), 243–255. 10.1016/B978-0-444-63784-0.00012-6

[B49] Kushi Y. Handa S. (1985). Direct Analysis of Lipids on Thin Layer Plates by Matrix-Assisted Secondary Ion Mass Spectrometry1. J. Biochem. 98, 265–268. 10.1093/oxfordjournals.jbchem.a135267 4044557

[B50] Leopold J. Popkova Y. Engel K. Schiller J. (2018). Recent Developments of Useful MALDI Matrices for the Mass Spectrometric Characterization of Lipids. Biomolecules 8, 173–198. 10.3390/biom8040173 PMC6316665 30551655

[B51] Lv Y. Lin Z. Tan T. Svec F. (2013). Preparation of Porous Styrenics-Based Monolithic Layers for Thin Layer Chromatography Coupled with Matrix-Assisted Laser-Desorption/ionization Time-Of-Flight Mass Spectrometric Detection. J. Chromatogr. A 1316, 154–159. 10.1016/j.chroma.2013.09.089 24128436

[B52] Makowska M. Pellinen T. (2021). Thin Layer Chromatography Performed in Stages to Identify the Presence of Aromatic like Fraction in Chosen Bitumen Modifiers. J. Traffic Transportation Eng. (English Edition) 8, 453–466. 10.1016/j.jtte.2019.09.008

[B53] Manikandan M. Kazibwe Z. Hasan N. Deenadayalan A. Gopal J. Pradeep T. (2016). Biological Desorption Electrospray Ionization Mass Spectrometry (DESI MS) - Unequivocal Role of Crucial Ionization Factors, Solvent System and Substrates. Trac Trends Anal. Chem. 78, 109–119. 10.1016/j.trac.2016.02.013

[B54] Mernie E. G. Tolesa L. D. Lee M.-J. Tseng M.-C. Chen Y.-J. (2019). Direct Oligosaccharide Profiling Using Thin-Layer Chromatography Coupled with Ionic Liquid-Stabilized Nanomatrix-Assisted Laser Desorption-Ionization Mass Spectrometry. Anal. Chem. 91, 11544–11552. 10.1021/acs.analchem.9b01241 31429260

[B55] Mirón-Mérida V. A. Wu M. Gong Y. Y. Guo Y. Holmes M. Ettelaie R. (2021). Mathematical Characterization of Ink Diffusion and Imbibition Processes in Chromatography Paper as a Potential Biosensing Platform. Sensing Bio-Sensing Res. 32, 100421. 10.1016/j.sbsr.2021.100421

[B56] Mohammad A. Khan M. Ullah Q. Mohammad F. (2017). Effective Separation of Organic Dyes Using Ionic Liquids as green mobile Phase and Polyaniline-Modified Silica Gel Nanocomposite-Based Thin-Layer Chromatography. J. Anal. Sci. Technol. 8, 1–14. 10.1186/s40543-017-0127-8

[B57] Móricz Á. M. Ott P. G. Yüce I. Darcsi A. Béni S. Morlock G. E. (2018). Effect-directed Analysis via Hyphenated High-Performance Thin-Layer Chromatography for Bioanalytical Profiling of sunflower Leaves. J. Chromatogr. A 1533, 213–220. 10.1016/j.chroma.2017.12.034 29269147

[B58] Morlock G. Schwack W. (2010). Coupling of Planar Chromatography to Mass Spectrometry. Trac Trends Anal. Chem. 29, 1157–1171. 10.1016/j.trac.2010.07.010

[B59] Morlock G. Ueda Y. (2007). New Coupling of Planar Chromatography with Direct Analysis in Real Time Mass Spectrometry. J. Chromatogr. A 1143, 243–251. 10.1016/j.chroma.2006.12.056 17223115

[B60] Ovcharov M. V. Barsegyan S. S. Kovaleva S. A. Kulikova L. N. Borisov R. S. (2017). New Approaches to the Application of DART Mass Spectrometry Coupled with Planar Chromatography for the Analysis of Mixtures of Organic Compounds. J. Anal. Chem. 72, 1446–1450. 10.1134/S106193481714009X

[B61] Peters V. Spangenberg B. (2019). Equol Determination in Cattle Manure by HPTLC-DART-TOF-MS. J. Liquid Chromatogr. Relat. Tech. 42, 311–316. 10.1080/10826076.2019.1585616

[B62] Qu J. Zhang Z.-H. Zhang H. Weng Z.-T. Wang J.-Y. (2021). Diethyl Malonate-Based Turn-On Chemical Probe for Detecting Hydrazine and its Bio-Imaging and Environmental Applications with Large Stokes Shift. Front. Chem. 8, 4457–4463. 10.3389/fchem.2020.602125 PMC8012553 33816431

[B63] Rejšek J. Vrkoslav V. Vaikkinen A. Haapala M. Kauppila T. J. Kostiainen R. (2016). Thin-Layer Chromatography/Desorption Atmospheric Pressure Photoionization Orbitrap Mass Spectrometry of Lipids. Anal. Chem. 88, 12279–12286. 10.1021/acs.analchem.6b03465 28193018

[B64] Rondeau D. (2017). “DART Mass Spectrometry: Principle and Ionization Facilities,” in Direct Analysis in Real Time Mass Spectrometry (Weinheim: Wiley-VCH Verlag GmbH & Co. KGaA), 43–80. 10.1002/9783527803705.ch2

[B65] Sahaka M. Amara S. Lecomte J. Rodier J.-D. Lafont D. Villeneuve P. (2021). Quantitative Monitoring of Galactolipid Hydrolysis by Pancreatic Lipase-Related Protein 2 Using Thin Layer Chromatography and Thymol-Sulfuric Acid Derivatization. J. Chromatogr. B 1173, 122674. 10.1016/j.jchromb.2021.122674 33827017

[B66] Sharma V. Kumar R. (2017). Fourier Transform Infrared Spectroscopy and High Performance Thin Layer Chromatography for Characterization and Multivariate Discrimination of Blue Ballpoint Pen Ink for Forensic Applications. Vibrational Spectrosc. 92, 96–104. 10.1016/j.vibspec.2017.05.006

[B67] Siebenhaller S. Gentes J. Infantes A. Muhle-Goll C. Kirschhöfer F. Brenner-Weiß G. (2018). Lipase-Catalyzed Synthesis of Sugar Esters in Honey and Agave Syrup. Front. Chem. 6, 1–9. 10.3389/fchem.2018.00024 29487847 PMC5816588

[B68] Speight J. (2020). Shale Oil and Gas Production Processes. Laramie, WY: CD&W Inc. 10.1016/B978-0-12-813315-6.00009-9

[B69] Stanek N. Kafarski P. Jasicka-Misiak I. (2019). Development of a High Performance Thin Layer Chromatography Method for the Rapid Qualification and Quantification of Phenolic Compounds and Abscisic Acid in Honeys. J. Chromatogr. A 1598, 209–215. 10.1016/j.chroma.2019.04.052 31023479

[B70] Taki T. (2015). TLC-blot (Far-Eastern Blot) and its Application to Functional Lipidomics. Methods Mol. Biol. 1314, 219–241. 10.1007/978-1-4939-2718-0_24 26139271

[B71] Torretta E. Fania C. Vasso M. Gelfi C. (2016). HPTLC-MALDI MS for (Glyco)sphingolipid Multiplexing in Tissues and Blood: A Promising Strategy for Biomarker Discovery and Clinical Applications. Electrophoresis 37, 2036–2049. 10.1002/elps.201600094 27162164

[B72] Van Berkel G. J. Ford M. J. Deibel M. A. (2005). Thin-Layer Chromatography and Mass Spectrometry Coupled Using Desorption Electrospray Ionization. Anal. Chem. 77, 1207–1215. 10.1021/ac048217p 15732898

[B73] Winter G. T. Wilhide J. A. LaCourse W. R. (2017). Analysis of Hop Acids by Thin-Layer Chromatography and the Molecular Ionization Desorption Analysis Source (MIDAS) for Mass Spectrometry. Int. J. Mass Spectrom. 422, 74–79. 10.1016/j.ijms.2017.08.013 26471042

[B74] Winter G. T. Wilhide J. A. LaCourse W. R. (2015). Molecular Ionization-Desorption Analysis Source (MIDAS) for Mass Spectrometry: Thin-Layer Chromatography. J. Am. Soc. Mass. Spectrom. 27, 352–358. 10.1007/s13361-015-1289-5 26471042

[B75] Zaikin V. G. Borisov R. S. (2021). Options of the Main Derivatization Approaches for Analytical ESI and MALDI Mass Spectrometry. Crit. Rev. Anal. Chem. 2021, 1–81. 10.1080/10408347.2021.1873100 33557614

[B76] Zaikin V. Halket J. (2009). A Handbook of Derivatives for Mass Spectrometry. Chichester: IM Publications.

[B77] Zarzycki P. K. (2015). Staining and Derivatization Techniques for Visualization in Planar Chromatography. Instrumental Thin-Layer Chromatogr. 2015, 191–237. 10.1016/B978-0-12-417223-4.00008-X

[B78] Zhang J. Zhou Z. Yang J. Zhang W. Bai Y. Liu H. (2012). Thin Layer Chromatography/Plasma Assisted Multiwavelength Laser Desorption Ionization Mass Spectrometry for Facile Separation and Selective Identification of Low Molecular Weight Compounds. Anal. Chem. 84, 1496–1503. 10.1021/ac202732y 22243032

[B79] Zhang N. Wang M. Li Y. Zhou M. Wu T. Cheng Z. (2020). TLC-MS Identification of Alkaloids in Leonuri Herba and Leonuri Fructus Aided by a Newly Developed Universal Derivatisation Reagent Optimised by the Response Surface Method. Phytochem. Anal. 32, 242–251. 10.1002/pca.2970 32559000

[B80] Zhang P. Zhang L. Shi J. Zhang N. Li Y. Wu T. (2019). TLC-electrostatic Field Induced spray Ionization-MS Analysis of Diverse Structural Skeletons and its Coupling with TLC Bioautography for Characterization of Lipase Inhibitory Components in American Ginseng. J. Pharm. Biomed. Anal. 174, 486–494. 10.1016/j.jpba.2019.06.019 31229845

[B81] Zhang Z. Ratnayaka S. N. Wirth M. J. (2011). Protein UTLC-MALDI-MS Using Thin Films of Submicrometer Silica Particles. J. Chromatogr. A 1218, 7196–7202. 10.1016/j.chroma.2011.07.098 21890140 PMC3196342

[B82] Zhong X. Qiao L. Liu B. Girault H. H. (2015). Ambient *In Situ* Analysis and Imaging of Both Hydrophilic and Hydrophobic Thin Layer Chromatography Plates by Electrostatic spray Ionization Mass Spectrometry. RSC Adv. 5, 75395–75402. 10.1039/C5RA10977A

